# Effects of coal-derived compound fertilizers on soil bacterial community structure in coal mining subsidence areas

**DOI:** 10.3389/fmicb.2023.1187572

**Published:** 2023-05-18

**Authors:** Huisheng Meng, Shuaibing Wang, Jie Zhang, Xiangying Wang, Chen Qiu, Jianping Hong

**Affiliations:** ^1^College of Resources and Environment, Shanxi Agricultural University, Taigu, Shanxi, China; ^2^College of Life Sciences, Shanxi Agricultural University, Taigu, Shanxi, China; ^3^College of Urban and Rural Construction, Shanxi Agricultural University, Taigu, Shanxi, China

**Keywords:** coal-derived compound fertilizer, soil reclamation, bacterial community, soil nutrients, fertilization treatment

## Abstract

The land damaged by coal mining can be recovered to healthy condition through various reclamation methods. Fertilization is one of the effective methods to improve soil fertility and microbial activity. However, the effects of coal-derived compound fertilizers (SH) on bacterial communities in coal mining subsidence areas still remain unclear. Here, we studied the effects on the nutrient characteristics and bacterial communities in fertilizer-reclaimed soil (CK, without fertilizer; CF, common compound fertilizers; SH, coal-derived compound fertilizers) in coal mining subsidence areas and we applied SH with four different nitrogen application rates (90, 135, 180, and 225 kg/hm^2^). The results showed that the application of SH significantly increased the contents of available nitrogen (AN), available phosphorus (AP), available potassium (AK), total phosphorus (TP) and soil organic matter (SOM) compared with CK, as well as the bacterial richness (Chao1) and diversity (Shannon) in reclaimed soil that increased first and then decreased with the increase of nitrogen application. Under the same nitrogen application rate (135 kg/hm^2^), the nutrient content, Chao1 and Shannon of SH2 treatments were higher than those of CF treatment. Meanwhile, SH increased the relative abundance of Proteobacteria, Actinobacteria and Gemmatimonadetes. LEfSe analysis indicated that the taxa of Acidobacteria and Actinobacteria were significantly improved under SH treatments. Canonical correspondence analysis (CCA) and Variance partitioning analysis (VPA) showed that SOM was the most important factor affecting the change of bacterial community structure in reclaimed soil. In conclusion, application of SH can not only increase nutrient content and bacterial diversity of reclaimed soil, but also improve bacterial community structure by increasing bacterial abundance.

## 1. Introduction

Coal is an important source of energy in the world, and China is the largest producer and consumer of coal in the world (Feng et al., 2019). Large-scale land subsidence due to coal mining occurs in many areas (Guo et al., 2018) and causes many environmental problems such as soil erosion, soil quality degradation, soil ecosystem imbalance, etc. (Kumar et al., 2015; Ahirwal and Maiti, 2016; de Quadros et al., 2016; Ma et al., 2019). At the same time, a large number of solid wastes produced in mining area, which cannot be degraded in a short time, brings potential danger to the surrounding environment. Moreover, there are also some problems such as poor soil structure and low fertility in mining area (Heneghan et al., 2008). And land reclamation is an effective strategy to solve the conflict between coal mining and land resource protection (Hu Z. et al., 2014), soil reclamation aims to restore the nutrient properties and try to recover the soil from mining area to original condition through a series of reclamation methods (Upadhyay et al., 2016). Based on engineering, chemical and other measures, the reclaimed soil can quickly evolve to mellow soil, at the same time contribute to the increase the content of soil nutrient and promote the growth of crops. Therefore, relatively high ecological and economic benefits can be achieved in a short period of time with proper management of conservation methods (Feng et al., 2019). However, traditional fertilization method is presently a common method of soil reclamation in mining area. And due to excessive use of fertilizer and nitrogen addition, fertilizer nutrient use efficiency decreases so that it is not conducive to the sustainable development of agriculture (Carvalho, 2017; Chen et al., 2018). Therefore, it is very important to restore the quality of reclaimed soil in mining area, and it has become the current direction of development to use compound fertilizer developed from industrial and mining wastes to restore the quality of reclaimed soil.

Actually, industrial and mining wastes are not friendly enough, which mainly cause the area of land resources decreased and environment pollution if the wastes without properly solution. However, it is of great significance to explore the utilization in agricultural production because of the special structure and good physical and chemical properties, as well as make more land resources available. More importantly, coal slime, weathered coal and other industrial and mining wastes can improve soil properties, which has been increasingly widely used as a fertilizer synergist in agriculture (Leita et al., 2009; DiDonato and Hatcher, 2017; Amoah-Antwi et al., 2021). However, directly to apply the slime and weathered coal will not significantly stimulate microbial activity even with the supplement of nitrogen fertilizer (Tran et al., 2015), and slime is prone to cause secondary pollution due to water loss. A previous study showed that the combination of coal-derived compound fertilizer and microbial fertilizer produced from solid wastes (weathered coal, coal slime, fly ash and coal gangue) had significant effects on soil fertility, crop growth and utilization efficiency of water and fertilizers (Guo et al., 2016). Liu et al. (2017) revealed that there were different effects on soil enzyme activity and crop yield between different amounts of coal-derived compound fertilizers. The best effect was obtained when the amount of coal-derived compound fertilizer was 4,115 kg/hm^2^, and the yield of maize was increased by 6.96% ~ 218.90% and the urease activity was increased by 49.20% ~ 98.61% compared with the other two amounts. In addition, Fan et al. (2020) found that adding humic acid to the coal-derived compound fertilizers was more conducive to the improvement of soil carbon pool management index compared with ordinary coal-derived compound fertilizers. Besides, it was found that 135 kg/hm^2^ of nitrogen application had the best effect on the improvement of soil organic carbon and carbon pool management index in the treatment of different nitrogen application rates. Soil microorganisms are the most active part of soil, which are involved in the formation of soil structure (Sun et al., 2016) and crucial to vegetation establishment, soil formation and nutrient transformation. Especially in the early stage of reclamation (Machulla et al., 2005; Li et al., 2013), the composition and distribution of soil microbial community are highly sensitive to the changes in soil environment (Liu et al., 2020), which may have a huge impact on ecosystem function (Wu et al., 2017). Microorganisms are positively related to the degradation and transformation of various pollutants (Gianfreda and Rao, 2004), playing an essential role in soil restoration of mining area. Soil microorganisms can be used as a standard to evaluate the success of reclamation (Feng et al., 2019). However, researches on coal-derived compound fertilizers mainly focus on the effects of soil fertility and physicochemical properties, and few researches have explored the effects of coal-derived compound fertilizers on reclaimed soil microorganisms. How coal-derived compound fertilizers affect reclaimed soil bacterial communities in mining areas is still poorly understood.

Humic acid has large inner surface, good adsorption, adhesion and colloid dispersion, which can effectively reduce water loss from slime. At the same time, the combination of humic acid and various inorganic fertilizers contributes to the improvement of soil physicochemical properties and bioactivity (Muhammad et al., 2019; De Corato, 2020), and can also improve soil quality and increase fertilizer utilization efficiency (Chen et al., 2017), promote crop yield and quality (Ahmad et al., 2018; Khan et al., 2018). Therefore, this research adopts the coal-derived compound fertilizer, which is made up of solid waste and humic acid from industrial and mining areas and common chemical fertilizer, and sets up four kinds of different nitrogen application rates at the same time, aiming to test the hypothesis that different nitrogen application rates of coal-derived compound fertilizers would affect soil nutrients, bacterial diversity and community composition. The purpose of this study was to determine (i) whether and how the coal-derived compound fertilizer affects soil properties and changes soil bacterial community structure; (ii) determine which bacterial groups will be significantly affected by coal-derived compound fertilizer; and (iii) which soil properties contribute to changes of soil bacterial diversity and community composition. It is expected to provide basis for reutilization of solid waste, rational fertilization and reclamation of soil, and effective guidance for ecological restoration of degraded soil in coal-mining subsidence areas.

## 2. Materials and methods

### 2.1. Study site and experimental design

The experimental area is located in Luojianggou Village, Wangqiao Town, Xiangyuan County, Shanxi Province (112°42′-113°14′E and 36°23′-36°44′N). It has a warm temperate continental monsoon with an average temperature of 8°C – 9°C, the temperature from July to September is the highest, with an average of 23.4°C, and the annual average precipitation is 532.8 mm. The frost-free period is about 166 days. Before the subsidence, the land was flat and the soil was fertile. It subsidenced and turned into poor dry land, and the productivity of the land decreased seriously. The soil type is calcareous cinnamon soil, the basic characters of which were as follows (Table 1).

**Table 1:** Basic properties of reclaimed soil.

AN (mg/kg)	AP (mg/kg)	AK (mg/kg)	TN (g/kg)	TP (g/kg)	SOM (g/kg)
26.88	3.42	90.12	0.28	0.41	3.76

AN, available nitrogen; AP, available phosphorus; AK, available potassium; TN, total nitrogen; TP, total phosphorus; SOM, soil organic matter.

The tested fertilizers include two kinds, common compound fertilizer (CF, N: urea (46%), P_2_O_5_: single superphosphate (16%), K_2_O: potassium sulfate (52%)): the mass ratio of N, P_2_O_5_ and K_2_O is 25:10:10, produced by Shanxi Yefeng Chemical Fertilizer Co., Ltd. Coal-derived compound fertilizer (SH) is made of coal slime, humic acid, urea, calcium superphosphate and potassium ore powder in a certain proportion. The mass ratio of N, P_2_O_5_ and K_2_O is 25:10:10, and the organic mass is 26.65%.

The field experiment included six treatments: CK (no fertilizer), conventional compound fertilizer (CF, N 135 kg/hm^2^) and four coal-derived compound fertilizer treatments with nitrogen application rate of 90 kg/hm^2^ (SH1), 135 kg/hm^2^ (SH2), 180 kg/hm^2^ (SH3), 225 kg/hm^2^ (SH4), the contents of the base fertilizer in treatments of CF was 540 kg/hm^2^, SH1–SH4, respectively, were 360, 540, 720, 900 kg/hm^2^. All treatments had 3 replicates, 100 m^2^ (10 m × 10 m) per plot, randomly arranged, 18 plots in total. The maize variety planted is Dafeng 30, produced by Shanxi Dafeng Seed Industry Co., Ltd. Its growing period is 150 days, the planting density is 60,000 plants per hectare, and there is no irrigation during the whole growing period. Fertilizer is applied as a base fertilizer to the soil once a year before sowing.

### 2.2. Sampling and physicochemical analysis

Soil samples were collected after the maize harvest on September 28th, 2021. A total of 18 soil samples (6 treatments × 3 replicates) were analyzed. Soil samples from each plot were collected at 5 sampling points at a depth of 0 ~ 20 cm and mixed to form a single sample. After the removal of visible plant residues and stones, each sample was divided into two parts: one was stored at −80°C for DNA extraction, the other part is air-dried, ground and sifted to remove debris for chemical property analysis. All the chemical properties were determined by routine methods (Bao, 2000). Soil organic matter (SOM) content was measured by the method of potassium dichromate oxidation (K_2_Cr_2_O_7_), and available nitrogen (AN) content was measured using the alkaline hydrolysis-diffusion method. The available phosphorus (AP) was extracted with 0.5 mol/L NaHCO_3_ solution before being assayed using the colorimetric molybdenum blue method. The available potassium (AK) was extracted using 1 mol/L NH_4_OAc solution before being assayed via flame photometry. Total phosphorus (TP) was digested with sulfuric acid-perchloric acid and then measured via molybdenum blue.

### 2.3. DNA extraction and PCR amplification

Total DNA was extracted from 0.25 g of each soil sample using the HiPure Soil DNA Kits (Magen, Guangzhou, China). DNA concentration and purity were determined using NanoDrop ND-2000 photometer (Thermo Scientific, Wilmington, DE, United States). The V3-V4 region of the 16S rRNA gene was amplified using paired primers 341F (5′-CCTACGGGNBGCASCAG-3′) and 806R (5′-GGACTACHVGGGTATCTAAT-3′). The PCR reaction conditions were as follows: 95°C predenaturation for 5 min; 95°C denaturation for 1 min, 60°C annealing for 1 min, 72°C extension for 1 min, 30 cycles; and final 7 min extension at 72°C. The gel-purified products were used to construct a library, and they were sequenced on an Illumina MiSeq platform (Gene Denovo Biotechnology Co., Ltd., Guangzhou, China).

### 2.4. Illumina MiSeq sequencing and sequence processing

After the 16S rRNA gene fragment was sequenced, the obtained reads were filtered to remove adaptor sequences, low-quality sequences, and sequences with more than 10% of unknown nucleotides (N). The chimeras were checked and removed using the UCHIME (Edgar et al., 2011). The clean reads were clustered into operational taxonomic units (OTUs) of  ≥  97% similarity using UPARSE (Edgar, 2013; version 9.2.64). The sequence with highest abundance was selected as representative sequence within each OTU. The representative OTU sequences were classified into organisms by a naive Bayesian model using RDP classifier (version 2.2) based on SILVA database (version 132),^1^ with the confidence threshold value of 1. BioProject number: PRJNA944927.

### 2.5. Statistical analysis

One-way analysis of variance (ANOVA), and Tukey’s HSD test were used to compare the means for each environmental variable, with a significance level of *p* < 0.05. Non-metric multidimensional scaling plots (NMDS) were used to evaluate the overall differences in bacterial community composition, based on the Bray-Curtis distances. Alpha Diversity Indices, including Shannon and Chao1, were calculated using Mothur (Schloss et al., 2009). Adonis test was used to analyze the explanatory degree of the sample difference, and the permutation test was used to analyze the statistical significance of the groups. The correlation between soil bacterial community structure and environmental factors was tested using Mantel’s test. Canonical correspondence analysis (CCA) was performed using the R project “Vegan” package (version 2.5.3) and Variance partitioning analysis (VPA) was based on the species and environment factor abundances table, with the envfit test used to assess the significance of the impact of each factor in the CCA model, in order to clarify the influence of environmental factors on community composition. Pearson correlation coefficients between environmental factors and alpha diversity were calculated using the R project “psych” package. To identify taxa which were significantly affected by fertilization, we carried out the linear discriminant analysis (LDA) effect size (LEfSe; Segata et al., 2011), biomarkers examined in this study are consistent with the following standards: (i) minimum LDA score (log10 value) for discriminative features are ≥3 and (ii) alpha value for the factorial Kruskal-Wallis test between groups ≤0.05.

## 3. Results

### 3.1. Changes of nutrient content in reclaimed soil

The results showed that the contents of AN, AP and TP were significantly increased after fertilization compared with CK (*p* < 0.05), and the overall order was SH > CF > CK (Table 2). While under SH2 treatment with the same nitrogen application rate, the results indicated that the effect of coal-derived compound fertilizer on soil nutrient content was better than that of common compound fertilizer. In particular, the contents of AN, AP, AK, SOM and TP in SH2 treatment were significantly increased by 17.46, 43.62, 24.43, 26.51, and 19.58% compared with CF (*p* < 0.05), respectively. In addition, the contents of AN, AP, AK, SOM and TP in SH treatments increased first and then decreased with the increase of nitrogen application, and the contents in SH3 treatment were the highest, respectively, increased by 58.46, 112.65, 35.00, 26.51, and 19.58%, which was significantly different from that in CF treatment (*p* < 0.05).

**Table 2:** Basic nutrient content of reclaimed soil.

Treatment	AN (mg/kg)	AP (mg/kg)	AK (mg/kg)	SOM (g/kg)	TP (g/kg)
CK	26.36 ± 2.10d	5.38 ± 0.45d	175.35 ± 5.55d	8.52 ± 0.23c	0.475 ± 0.018d
CF	35.80 ± 1.26c	9.17 ± 0.76c	187.83 ± 2.66 cd	9.92 ± 0.38bc	0.526 ± 0.010c
SH1	40.02 ± 4.37b	12.33 ± 1.26b	197.57 ± 3.02c	11.33 ± 0.81ab	0.561 ± 0.040c
SH2	42.05 ± 3.41b	13.17 ± 0.76b	233.72 ± 3.36b	12.06 ± 0.82a	0.582 ± 0.016b
SH3	56.73 ± 4.35a	19.50 ± 0.50a	253.57 ± 3.24a	12.55 ± 0.65a	0.629 ± 0.021a
SH4	53.32 ± 2.34a	18.83 ± 1.26a	247.39 ± 4.82a	11.83 ± 0.25ab	0.611 ± 0.019a

Significance among treatments was tested using one-way ANOVA at *p* < 0.05. Different letters in a single row indicate a significant difference between treatments. AN, available nitrogen; AP, available phosphorus; AK, available potassium; SOM, soil organic matter; TP, total phosphorus.

### 3.2. Bacterial alpha diversity under different fertilization

**Figure 1:**
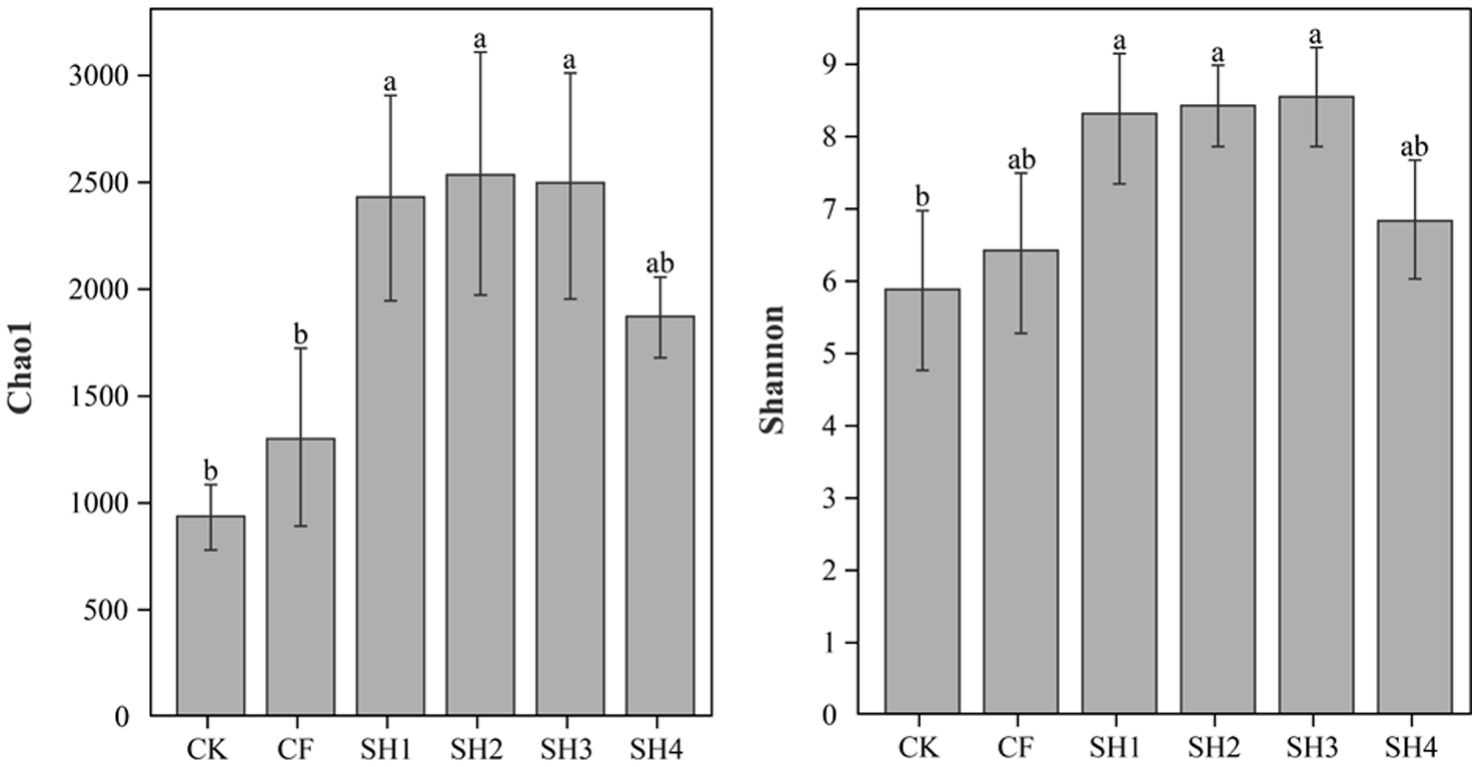
Comparison of estimated alpha diversity indices of 16 s rRNA gene libraries for clustering at 97% similarity, obtained from high throughput sequencing analysis. Data are the means, *n* = 4, error bars represent standard error. Bars with the same letter are not significantly different at *p* ≤ 0.05.

Bacterial diversity and richness (Figure 1). Compared with CK, fertilization could effectively improve bacterial richness (Chao1) and diversity (Shannon) in reclaimed soil. At the same nitrogen application rate, Chao1 in SH2 was significantly higher than that in CF (*p* < 0.05), and Shannon in SH2 was higher than that in CF though not significantly. In addition, Chao1 and Shannon in SH treatments increased initially and decreased afterwards with the increase of nitrogen application. Specifically, Chao1 was highest in SH2 treatment while Shannon in SH3 treatment.

### 3.3. Composition of soil bacterial communities

**Figure 2:**
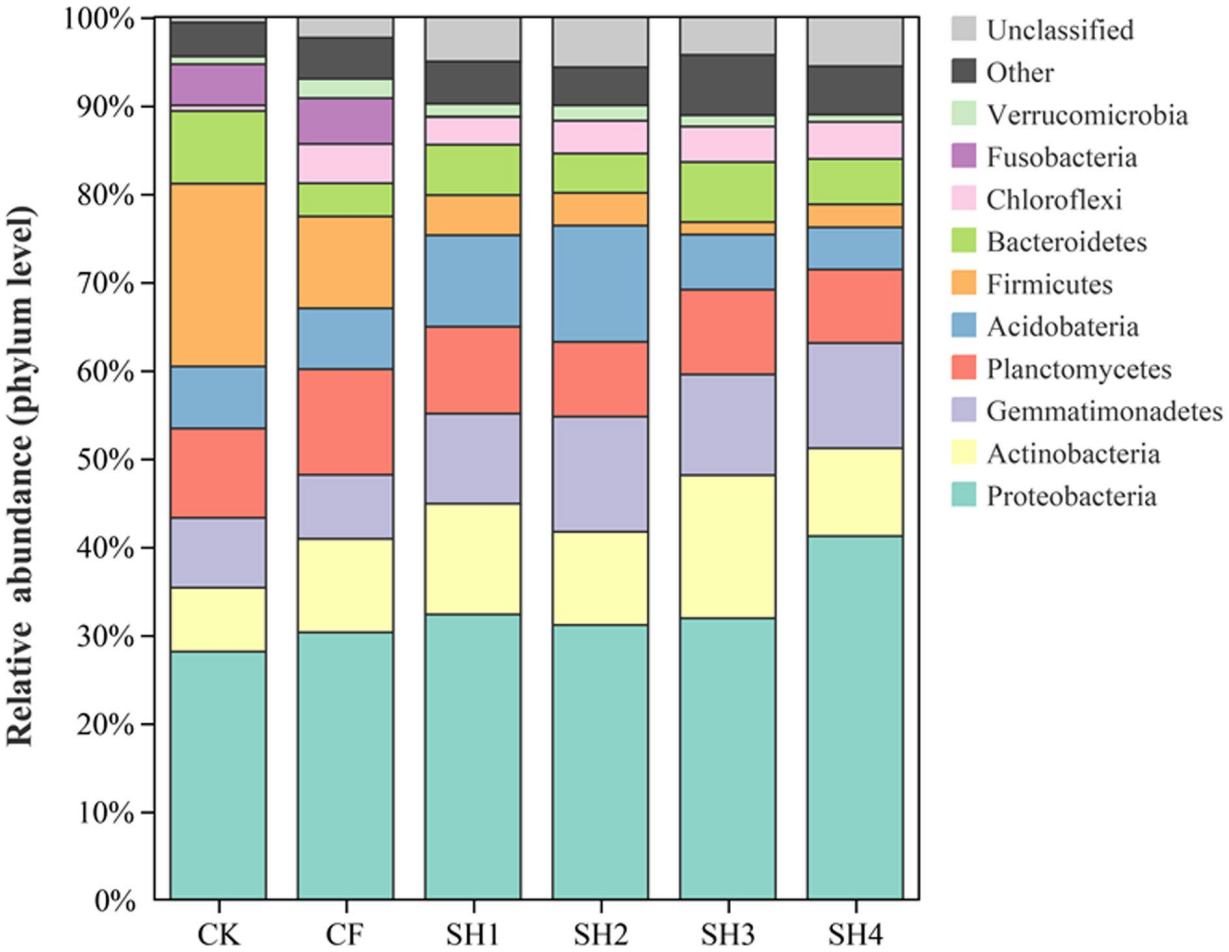
Relative abundance of species at phylum level.

We evaluated the taxonomic composition of bacteria, with Proteobacteria, Actinobacteria, Gemmatimonadetes, Planctomycetes, and Acidobacteria being the dominant phyla in reclaimed soil (Figure 2). Compared with CK, the relative abundance of Proteobacteria, Actinobacteria and Gemmatimonadetes were increased in SH treatments, while the relative abundance of Planctomycetes was decreased in SH treatments. And the highest relative abundance of Proteobacteria was detected in SH4 treatment (Supplementary Table S1), which was significantly different from CK (*p* < 0.05). Besides, the relative abundance of Gemmatimonadetes and Acidobacteria were highest in SH2 treatment (*p* < 0.05) and the highest relative abundance of Actinobacteria was found in SH3 all significantly different from CK (*p* < 0.05). CF treatment increased the relative abundance of Proteobacteria, Actinobacteria and Planctomycetes, while decreased the abundance of Gemmatimonadetes and Acidobacteria.

**Figure 3:**
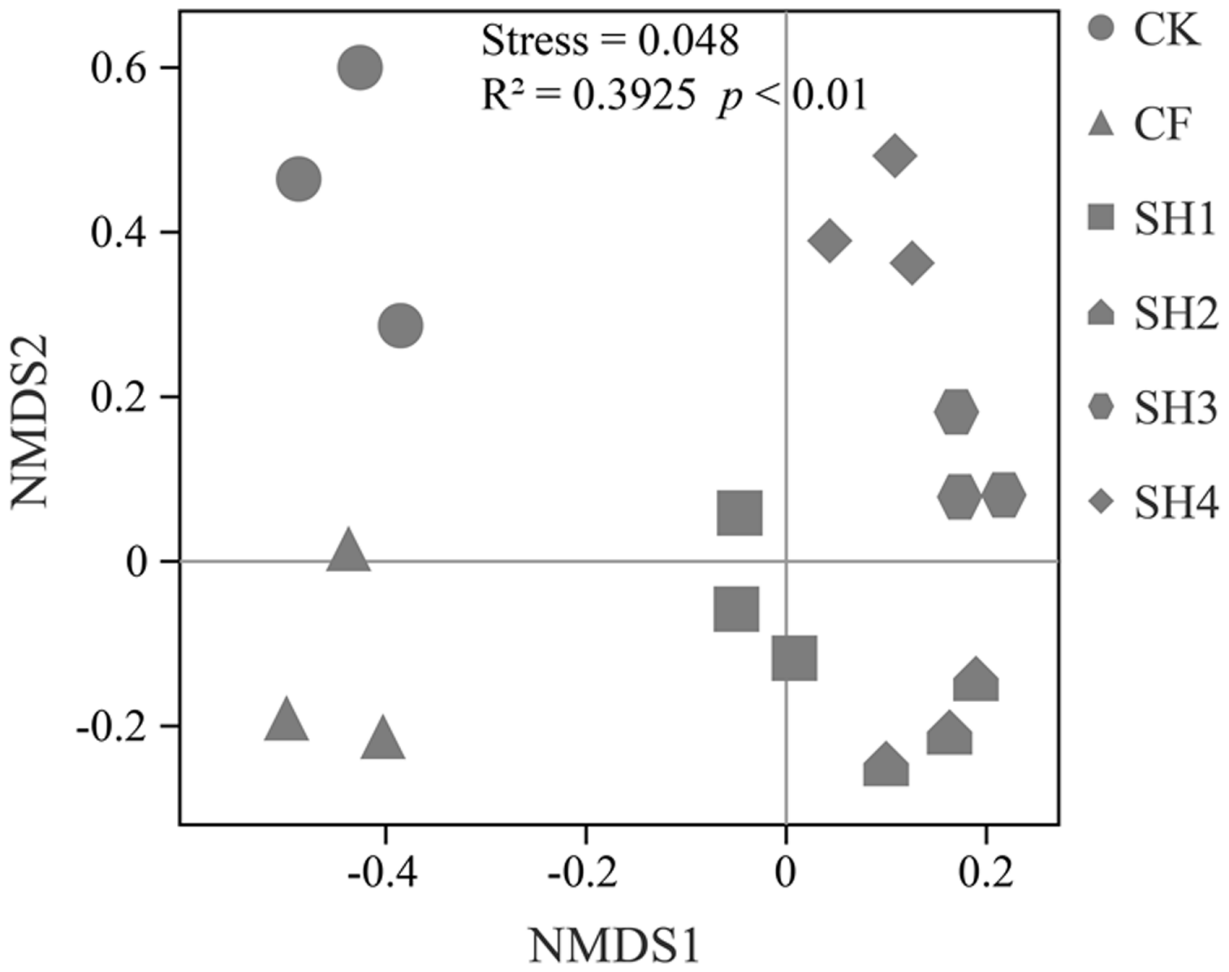
Effects of fertilization on bacterial communities composition (Genus level) in reclaimed soil. Non-metric multidimensional scaling (NMDS) of bacterial communities based on Bray-Curtis dissimilarities.

To determine whether fertilization led to shifts in soil bacterial communities, we profiled the structural changes in bacterial communities (Genus level) using NMDS based on Bray-Curtis dissimilarities (Figure 3). There were significant differences between bacterial communities and CK in different fertilization treatments, and the stress of 0.048 also indicated that NMDS fit well. There were also significant differences among the four SH nitrogen application rates. In addition, Adonis (Permanova) test confirmed that there was significant difference in soil microbial community among different treatments (*R*^2^ = 0.3925, *p* < 0.01).

**Figure 4:**
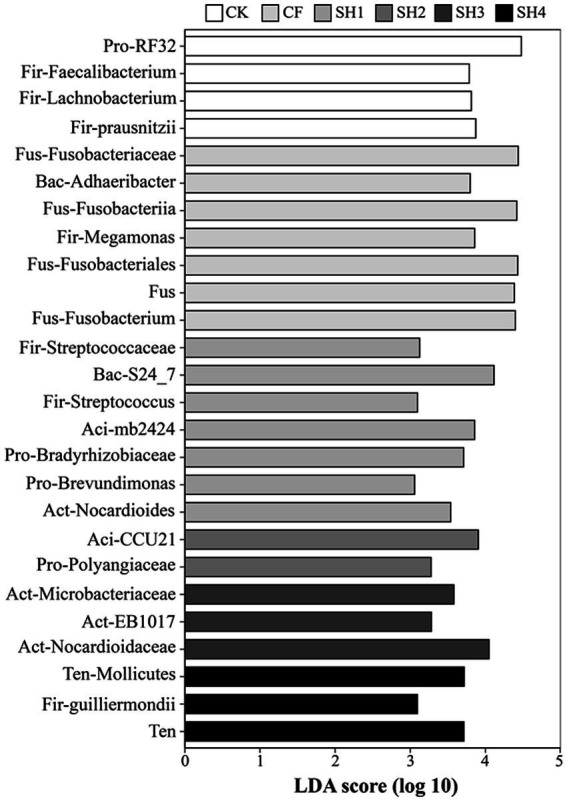
In reclaimed soils, key phylogenetic phenotypes of bacteria were significantly altered between treatments using linear discriminant analysis (LDA) effect sizes (LEfSe). Pro, Proteobacteria; Fir, Firmicutes; Fus, Fusobacteria; Bac, Bacteroidetes; Aci, Acidobacteria; Act, Actinobacteria; Ten, Tenericutes; Aci, Acidobacteria; Act, Actinobacteria; Ten, Tenericutes.

We further identified high-dimensional biomarkers at each taxonomic level using LEfSe and explored the significantly changed taxa (Figure 4). The results showed that the taxa of RF32 (LDA score = 4.48) changed significantly in CK. However, Bradyrhizobiaceae (LDA score = 3.71) and *Brevundimonas* (LDA score = 3.06) were significantly altered in SH1, while Polyangiaceae (LDA score = 3.27) was significantly altered in SH2. In addition, we found that the abundance of Firmicutes and Fusobacteria was the highest in CK and CF treatments, and only three taxa from Firmicutes were significantly decreased in SH1 and SH4 treatments including Streptococcaceae (LDA score = 3.12), *Streptococcus* (LDA score = 3.10), and Guilliermondii (LDA score = 3.09). Meanwhile, the Acidobacteria taxa and Actinobacteria taxa were only found in SH treatments.

### 3.4. Relationship between bacterial diversity and community composition with soil properties

**Figure 5:**
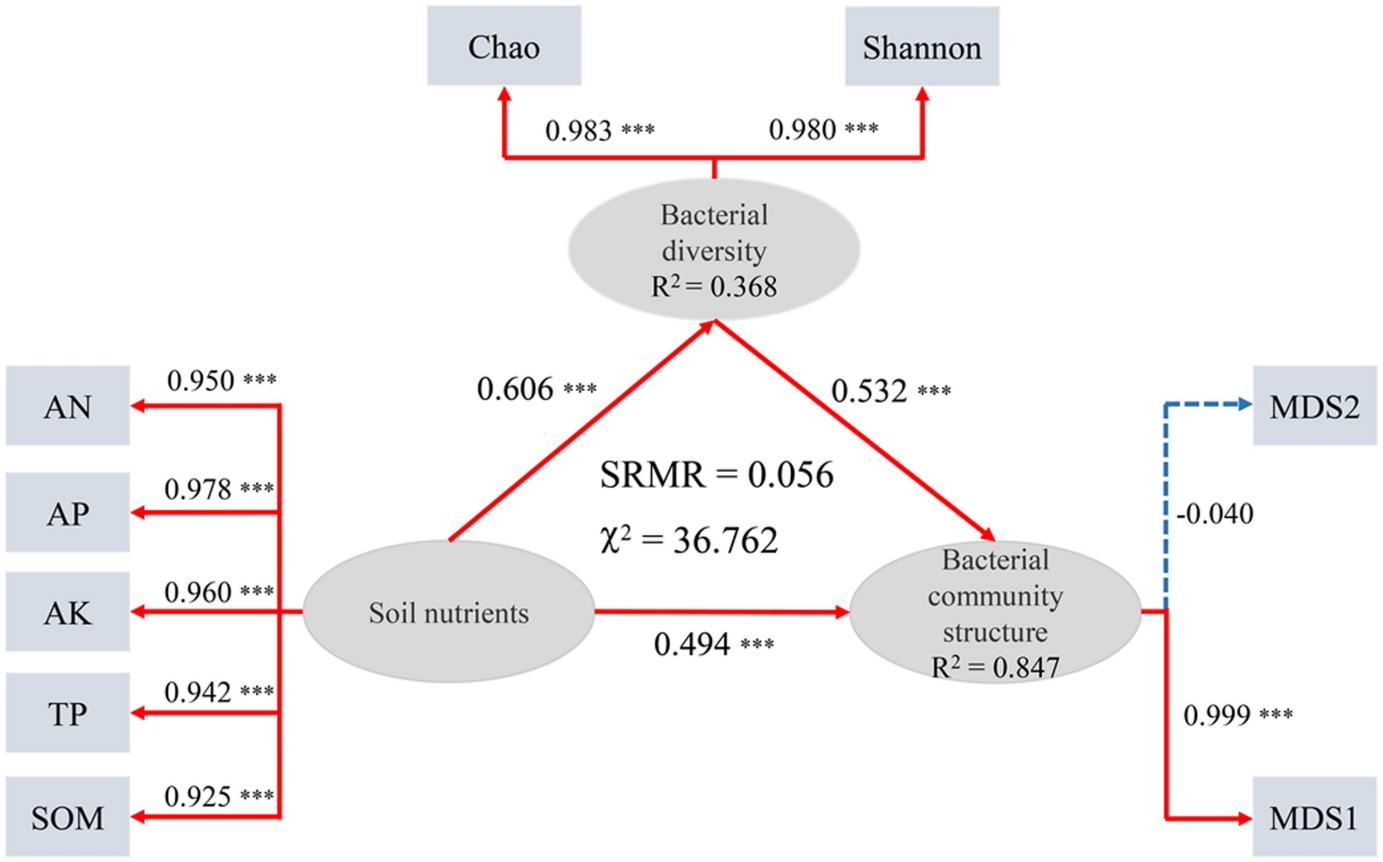
Partial Least Squares Path Modeling (PLSPM). Each oval shape represents an observed variable (i.e., measured) and box represents latent variable (i.e., constructs). Red lines represent positive effects and blue lines represent negative effect. Dashed arrows show that coefficients did not differ significantly from 0 (*p* > 0.05). Numbers on the lines in the PLSPM model are the ‘total effects’ values, ***p < 0.001.

The Mantel test for the association between bacterial community structure (Bray-Curtis distance) and soil properties indicated that AN, AP, TP, and SOM were significantly correlated with bacterial community structure (*p* < 0.05, Supplementary Table S2). The relationship among soil nutrients, bacterial diversity and bacterial community structure was determined using the PLSPM (Figure 5). The results of the PLSPM revealed that soil nutrients had significant positive correlation with bacterial diversity (0.606) and bacterial community structure (0.494, *p* < 0.001). Bacterial diversity had significant positive relationships with bacterial community structure (0.532, *p* < 0.001). Meanwhile, soil nutrients can also affect the bacterial community structure indirectly through soil microbial diversity.

**Figure 6:**
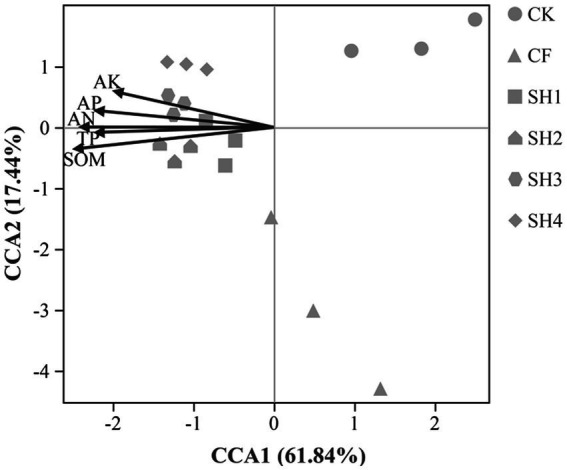
Canonical correspondence analysis indicated the potential relationship between soil environmental variables and soil bacterial community (Genus level).

**Figure 7:**
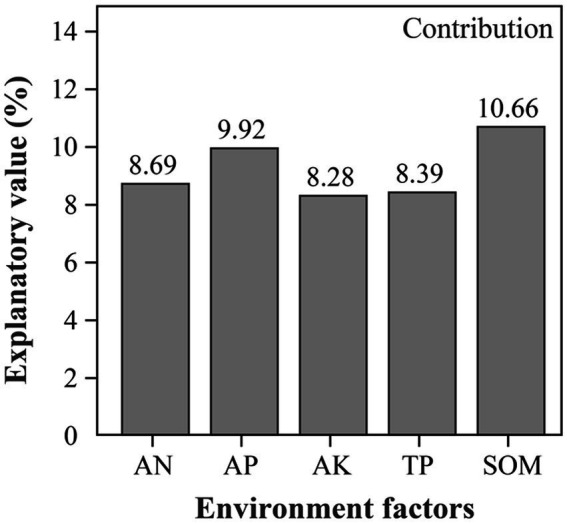
VPA analysis of the contribution of environmental factors to the total variation of species.

CCA results directly show the effect of environmental factors on soil bacterial communities (Figure 6). At the Genus level, the five soil parameters accounted for 79.82% of the total variation, with the first and second axes, respectively, accounting for 61.84 and 17.44%, respectively. The bacterial communities in soil from SH treatments were positively correlated with the concentrations of AN, AP, AK, TP and SOM. Among these, SOM (*R*^2^ = 0.6985, *p* = 0.001) was the most important environmental factor affecting the variation of bacterial community. Besides, variance partitioning analysis (VPA) showed that SOM accounted for 10.66% of the total Variance and followed by AP 9.92% (Figure 7), which was further verified that SOM was the most important factor affecting the variation of bacterial community structure in reclaimed soil. In addition, AN, AP, AK and TP were also significantly correlated with the variance of bacterial community (Supplementary Table S3).

## 4. Discussion

### 4.1. Effects of coal-derived compound fertilizers on soil properties and alpha diversity of bacteria

Fertilization can effectively improve the soil’s total and available nutrients, and accelerate soil maturity (Sun et al., 2016). Excessive use of chemical fertilizers hardens the soil, reduces soil fertility and pollutes the natural environment (Pahalvi et al., 2021). Low-rank coals and their derivatives are rich in micronutrients and are also valuable sources of organic matter containing large amounts of humus (Akimbekov et al., 2021), which can be used as an alternative source for the management of soil fertility. During our reclamation, coal-derived compound fertilizers significantly increased soil nutrients (AN, AP, AK, SOM, and TP; Table 2), especially in SH3 treatment with 180 kg/hm^2^ nitrogen application, and the results were similar to those in this study (Li et al., 2019; Dehsheikh et al., 2020). In addition, SH2 had a better effect on soil nutrient content than CF under the same nitrogen application rate (135 kg/hm^2^), which may be due to the addition of humic acid to coal-derived compound fertilizer. The results indicated that the combination of humic acid and inorganic fertilizer can effectively improve the utilization rate of fertilizer (Chen et al., 2017). It is worth noting that when the nitrogen application rate increased to 225 kg/hm^2^ (SH4), soil nutrient content decreased in different degrees, which indicated that the effect of combined application of coal-derived compound fertilizer and suitable nitrogen amount on soil nutrient was better, and excessive nitrogen application had a negative effect on SOM synthesis and decomposition (Galloway et al., 2008), while soil acidification caused by excess nitrogen will prevent inorganic phosphorus from adsorbing to mineral surfaces (Cusack et al., 2016). Reducing the amount of chemical fertilizer combined application is more beneficial to the growth of soil nutrient level (Zhong et al., 2010; Zhao et al., 2016).

Soil microbial diversity is critical to the integrity, function and long-term sustainability of soil ecosystems (Kandeler et al., 2006; Cheng et al., 2018), and restoration of microbial diversity is a key issue in regenerating soil systems. In this study, we analyzed microbial diversity and richness using Shannon and Chao1, respectively. The results showed that SH could effectively increase the bacteria alpha diversity bacteria compared with CK treatment. At the same nitrogen rate (135 kg/hm^2^), the bacteria alpha diversity in SH2 treatment was higher than that in CF treatment, which indicated that the application of coal-derived compound fertilizer promoted the recovery of soil microorganisms (Figure 1). Interestingly, Chao1 and Shannon also showed a trend of first increasing and then decreasing with the increase of nitrogen application in SH, which is consistent with the trend of nutrient content change in reclaimed soil, there is ample evidence that an increase in nitrogen input leads to a decrease in bacterial diversity (Zhou et al., 2015), and that high mineral nitrogen content may create stress conditions that inhibit bacterial growth, resulting in a significant decrease in bacterial diversity (Shen et al., 2016). Moreover, the subsidy-stress hypothesis states that higher levels of nitrogen addition results in reduced diversity, while moderate levels of amendment can promote microbial diversity (Odum et al., 1979; Odum, 1985). The application of exogenous organic matter is often crucial to improve soil fertility and nutrient management. The addition of humic acids to inorganic fertilizers can change soil microbial community structure, improve soil microbial diversity and soil nutrient content (Li et al., 2019; Wang et al., 2022), which were similar to the results of this experiment. To sum up, coal-derived compound fertilizer can effectively improve soil nutrients and promote the recovery of soil microbial diversity, and SH3 (180 kg/hm^2^ nitrogen application) is the most suitable.

### 4.2. Effect of coal-derived compound fertilizer on bacterial community structure

NMDS and Adonis (Permanova) test showed that fertilization resulted in significant differences in soil bacterial community composition, and there were also significant differences in SH among four nitrogen application rates (Figure 2). According to the analysis of the changes in taxa composition (Figure 3), the relative abundance of Proteobacteria in the reclaimed soil showed an overall increasing trend with the increase of SH nitrogen application, while Acidobacteria decreased with the increase of SH nitrogen application (Supplementary Table S1), which are consistent with previous studies (Ling et al., 2017; Wang et al., 2018). The changes in bacterial composition after the increase of nitrogen application can be explained by the eutrophication hypothesis (Fierer et al., 2007). And Proteobacteria can adapt to resource-rich (carbon-rich) environments well. Moreover, as a co-trophic group, Proteobacteria has a rapid growth rate and is more likely to grow under nutrient-rich conditions (Fierer et al., 2007, 2012), while nutrient-poor populations are conducive to growth under low-carbon conditions.

In the study, Proteobacteria, Actinobacteria and Acidobacteria were the main phylums from reclaimed soil in mining area, which was consistent with other researches about soil microbial community. Among these, Proteobacteria was the key phylums with the highest relative abundance in reclaimed soil, due to it could survive in extreme environment via carbon cycling and nitrogen fixing processes (Kasemodel et al., 2019). We found that soil nutrient contents (AN, AP, AK, TP, and SOM) were positively correlated with the abundance of Proteobacteria, Actinobacteria and Gemmatimonadetes, while negatively correlated with the abundance of Firmicutes and Fusobacteria (Supplementary Figure S1). The LEfSe analysis also showed that Actinobacteria taxa were only found in the treatments with high soil nutrient content (SH1 – SH3; Figure 4), indicating that *Nocardioides*, Microbacteriaceae, EB1017, Nocardioidaceae were positively correlated with soil nutrient contents. Studies have shown that Actinobacteria plays an important role in improving agricultural soil quality (Francioli et al., 2016), participates in organic matter turnover and carbon cycling, supplements the nutrient supply in soils and is an important component of humus formation (Anandan et al., 2016). In this study, *Nocardioides*, Microbacteriaceae, and Nocardioidaceae are all belong to Actinomycetales. Previous reports have shown that members of Actinomycetales have many ecological roles, such as the potential to inhibit the growth of a variety of plant pathogens (Sprusansky et al., 2005; Jeffrey et al., 2007), to promote plant growth, and it also can help to solubilize phosphate, produce siderophore and fix nitrogen (Bhatti et al., 2017). In addition, Actinomycetes do not pollute the environment; on the contrary, they help maintain a biological balance between soil and nutrient cycling. However, the taxa of Firmicutes and Fusobacteria were only found in CK, CF and SH1 with low nitrogen application, indicating that the taxa of Firmicutes and Fusobacteria were negatively correlated with soil nutrient content. Our results suggest that Proteobacteria and Actinobacteria are the dominant phyla in reclaimed soils (Figure 3), which is consistent with previous studies (Lauber et al., 2009; Rastogi et al., 2010; Zhan and Sun, 2014). The consistency of dominant phyla in different mining areas shows that these bacteria play an important role in soil improvement and have extensive adaptability to soil environment in mining areas.

### 4.3. Relationship between soil properties versus bacterial diversity and community structure

The interactions between soil chemical properties, biological properties, and microbial communities have triggered debates about the mechanisms of nutrient cycling and ecosystem processes, and understanding these complex interactions is essential for the proper functioning of biomes. Numerous soil properties play critical roles in shaping bacterial community structure and diversity when the soil is subjected to fertilization (Dangi et al., 2012; Sun et al., 2016; Yu et al., 2019), and improving soil fertility leads to higher bacterial abundance and diversity (Cui et al., 2012; Li et al., 2014). In this study, we found that Chao1 and Shannon were positively correlated with SOM, AP and TP (*p* < 0.001, *p* < 0.01, and *p* < 0.05), and the results showed that the accumulation of SOM and nutrients could well explain the higher bacterial richness and diversity index in reclaimed soil. We also found that most abundant phyla were significantly associated with one or more selected soil properties, underscoring the critical role of SOM and nutrients in shaping the abundance and diversity of soil bacterial communities (Supplementary Figure S1).

Previous studies have shown that SOM and AP are major factors influencing the composition of bacterial communities in soil (Fierer et al., 2012; Hu Y. et al., 2014; Liu et al., 2014; Yu et al., 2019), which is consistent with our study. For one thing, the reclaimed soil is exposed to poor soil nutrients and low SOM content. While the content of SOM influence ecosystem function more, it would induce the change of bacterial community structure if increased SOM in a short time (Zhang et al., 2023). For another thing, SOM can effectively promote the growth and recovery of bacteria in reclaimed soil, while fertilization can apparently improve the content of SOM and apply energy for the activities of microorganisms, which contribute to the increase of soil microbial diversity and influence the composition of bacterial community. In this study, the results of CCA and Mantel test indicated that SOM was the main factor influencing bacterial community structure in reclaimed soil (Figure 6; Supplementary Table S2). Many biological factors directly affect carbon mineralization in soils, and carbon sources are one of the most important factors affecting microbial communities (Blagodatskaya and Kuzyakov, 2008). VPA and correlation analysis further showed that SOM had a positive effect on soil microbial diversity (Figure 7). Studies have shown that the increase of SOM can promote soil agglomeration, improve soil physical properties and nutrients, thereby promoting the growth and recovery of bacteria in reclaimed soil (Li L. et al., 2021). Meanwhile, SOM plays a key role in plant growth and directly affects soil bacterial diversity (Zeng et al., 2016; Li W. et al., 2021; Xu et al., 2021). Moreover, as a reservoir of carbon and nitrogen sources, continuous decomposition of SOM can contribute to the improvement of bacterial community diversity by producing multiple substrates for the microbiota (Li et al., 2014).

The purpose of soil reclamation in coal mining subsidence area is to reconstruct productive, healthy and sustainable ecosystem of land use after mining. Increasing soil nutrients and enriching microbial population through fertilization is an effective method for ecological restoration in mining areas (Mummey et al., 2002; Cao et al., 2020; Li et al., 2020; Yan et al., 2021). In our study, soil nutrient and bacterial community diversity increased significantly after 4 years of reclamation. However, the reconstruction of soil microbial community in coal mining subsidence area depends not only on the mode of reclamation, but also on the time of reclamation. The study on the reclamation of mining area shows that the most important restoration stage of microbial community is 5 ~ 20 years after reclamation, and the difference is mainly related to fertilization, soil properties and vegetation (Dangi et al., 2012; Li et al., 2015; Cheng et al., 2018). Therefore, there is still a need for continuous monitoring of soil nutrients and microbial communities in selected sites, taking into account the effects of fertilization and the timing of reclamation.

## 5. Conclusion

In conclusion, the application of coal-deriver compound fertilizer can promote soil bacterial diversity and community composition by improving soil nutrients, and improve beneficial bacterial groups that play a significant role in ecosystem function. After 4 years of reclamation, the abundance and diversity of soil bacteria increased with the increase of soil nutrients. In addition, fertilization could effectively increase the abundance of soil bacteria, and it is more obvious that coal-derived compound fertilizer could positively affect bacterial diversity, especially SOM, which is critical for the formation of major bacterial populations.

## Data availability statement

The original contributions presented in the study are included in the article/Supplementary material, further inquiries can be directed to the corresponding author.

## Author contributions

HM provided the resources for performing the experiment, and helped to came up with the idea for this article. SW was responsible for the experiment process, data curation, original draft writing, and article revised. JZ, XW, CQ, and JH contributed to review and editing of manuscript. All authors contributed to manuscript revision, read, and approved the submitted version.

## Funding

This work was supported by the State Key Program of National Natural Science of China (Grant number U1710255-3), National Natural Science Foundation of China (41907215), Natural Science Research Project of Shanxi Province (202103021224171) and Technology Innovation Fund of Shanxi Agricultural University (2017ZZ08).

## Conflict of interest

The authors declare that the research was conducted in the absence of any commercial or financial relationships that could be construed as a potential conflict of interest.

## Publisher’s note

All claims expressed in this article are solely those of the authors and do not necessarily represent those of their affiliated organizations, or those of the publisher, the editors and the reviewers. Any product that may be evaluated in this article, or claim that may be made by its manufacturer, is not guaranteed or endorsed by the publisher.

## Footnotes

^1^ https://www.arb-silva.de/
