# Supplementary material for: Effects of coal-derived compound fertilizers on soil bacterial community structure in coal mining subsidence areas
Source: Front Microbiol. 2023 May 18;14:1187572. doi: 10.3389/fmicb.2023.1187572 (PMC10233127; doi:10.3389/fmicb.2023.1187572)
Supplement: Supplementary file 1 [file Data_Sheet_1.docx]

Supplementary Material

Effects of Coal-derived Compound Fertilizers on soil bacterial community structure in coal mining subsidence areas

Huisheng Meng^1†*^, Shuaibing Wang^1†^, Jie Zhang^1^, Xiangying Wang^2^, Chen Qiu^3^, Jianping Hong^1^

*** Correspondence:** Huisheng Meng: menghuisheng@sxau.edu.cn

# Supplementary Figures and Tables

## Supplementary Tables

Table. S1. Relative abundance of species at phylum level (top10).

| Phylum  Percentage (%) | Treatment |  |  |  |  |  |
| --- | --- | --- | --- | --- | --- | --- |
|  | CK | CF | SH1 | SH2 | SH3 | SH4 |
| Proteobacteria | 28.11b | 30.29b | 32.31ab | 31.12ab | 31.89ab | 41.19a |
| Actinobacteria | 7.24b | 10.59ab | 12.56ab | 10.56ab | 16.21a | 9.96ab |
| Gemmatimonadetes | 7.91b | 7.25ab | 10.20ab | 13.05a | 11.41a | 11.91a |
| Planctomycetes | 10.13a | 11.98a | 9.85a | 8.47a | 9.62a | 8.33a |
| Acidobacteria | 7.02b | 6.89b | 10.36ab | 13.18a | 6.23b | 4.78b |
| Firmicutes | 20.71a | 10.40a | 4.55a | 3.71a | 1.42a | 2.60a |
| Bacteroidetes | 8.24a | 3.76a | 5.71a | 4.46a | 6.81a | 5.15a |
| Chloroflexi | 0.64b | 4.45a | 3.14ab | 3.71a | 4.01a | 4.19a |
| Fusobacteria | 4.66ab | 5.19a | 0.06b | 0.00b | 0.00b | 0.00b |
| Verrucomicrobia | 0.88a | 2.18a | 1.44a | 1.75a | 1.29a | 0.82a |

Signiﬁcance among treatments was tested using one-way ANOVA at *p* < 0.05. Diﬀerent letters in a single row indicate a signiﬁcant diﬀerence between treatments.

Table. S2. Mantel test of the bacterial community structure (Bray-Curtis distance) with the soil properties.

| Properties | r | p |
| --- | --- | --- |
| AN | 0.508 | 0.001 |
| AP | 0.613 | 0.001 |
| AK | 0.373 | 0.001 |
| TP | 0.375 | 0.008 |
| SOM | 0.470 | 0.007 |

*p* values based on 999 permutations. AN: available nitrogen; AP: available phosphorus; AK: available potassium; TP: total phosphorus; SOM: soil organic matter.

Table. S3. The correlation between environmental factors and Genus was analyzed by Envfit test.

| Constrain | Envfit_R^2^ | Envfit_P |
| --- | --- | --- |
| AN | 0.6495 | 0.002 |
| AP | 0.6604 | 0.001 |
| AK | 0.6123 | 0.002 |
| TP | 0.5621 | 0.004 |
| SOM | 0.6985 | 0.001 |

## Supplementary Figure


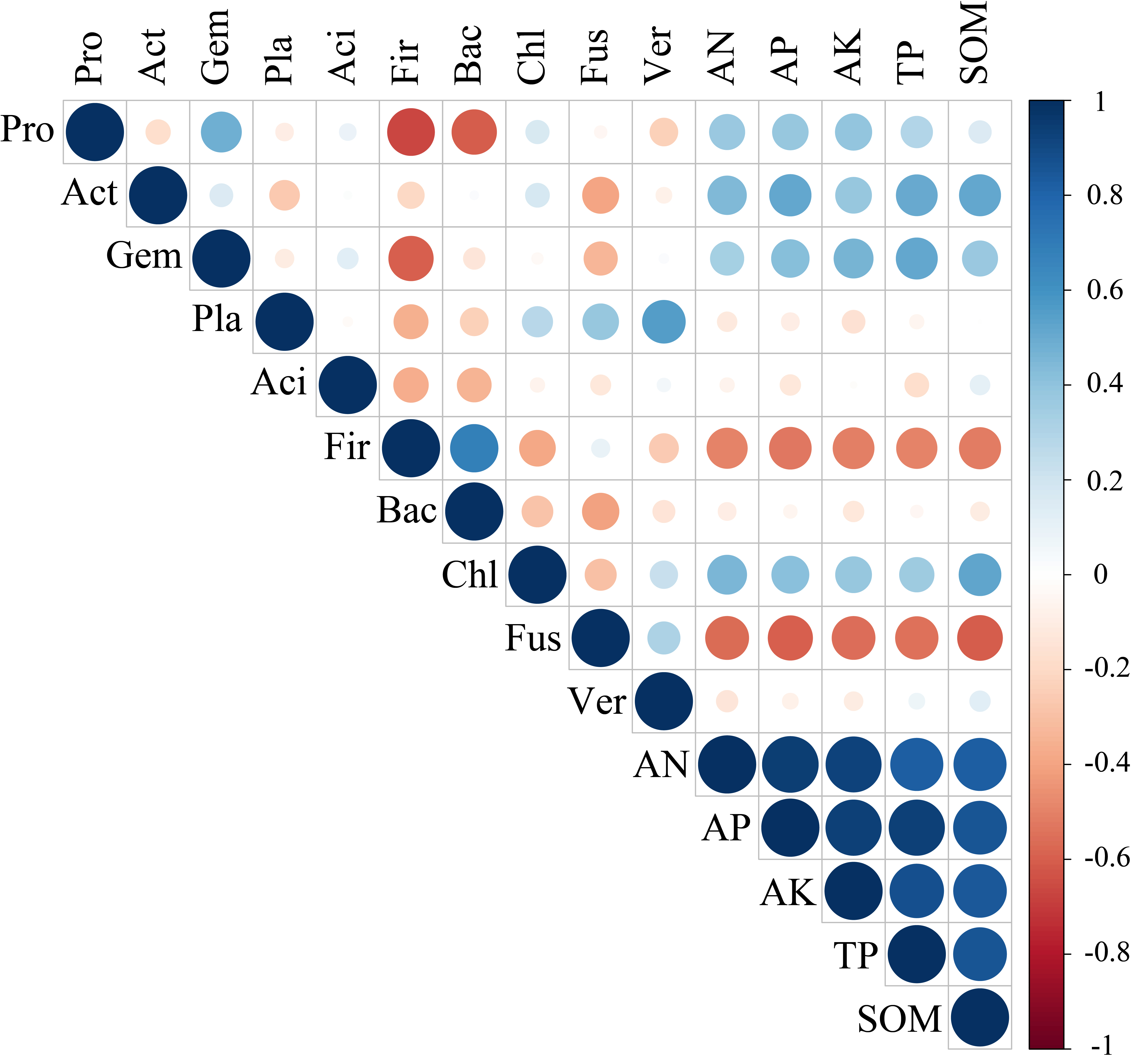


Fig. S1. Pearson correlation analysis between soil properties and most abundant bacterial phyla. Red and blue circles represent negative and positive correlation, respectively. Pro = Proteobacteria; Act = Actinobacteria; Gem = Gemmatimonadetes; Pla = Planctomycetes; Aci = Acidobacteria; Fir = Firmicutes; Bac = Bacteroidetes; Chl = Chloroflexi; Fus = Fusobacteria; Ver = Verrucomicrobia.
